# Egg White Alginate as a Novel Scaffold Biomaterial for 3D Salivary Cell Culturing

**DOI:** 10.3390/biomimetics7010005

**Published:** 2021-12-28

**Authors:** Hieu M. Pham, Yuli Zhang, Jose G. Munguia-Lopez, Simon D. Tran

**Affiliations:** 1 Faculty of Dentistry, McGill University, 3640 University Street, Montreal, QC H3A 0C7, Canada; mikehieupham@hotmail.com (H.M.P.); yuli.zhang@mail.mcgill.ca (Y.Z.); jose.munguia-lopez@mail.mcgill.ca (J.G.M.-L.); 2 College of Dentistry, New York University, 345 E 24th Street, New York, NY 10010, USA; 3 Department of Bioengineering, McGill University, 3480 University Street, Montreal, QC H3A 0E9, Canada

**Keywords:** tissue engineering, hydrogel, egg white, alginate, Matrigel, salivary tissue culture

## Abstract

Saliva production by salivary glands play a crucial role in oral health. The loss of salivary gland function could lead to xerostomia, a condition also known as dry mouth. Significant reduction in saliva production could lead to further complications such as difficulty in speech, mastication, and increased susceptibility to dental caries and oral infections and diseases. While some palliative treatments are available for xerostomia, there are no curative treatments to date. This study explores the use of Egg White Alginate (EWA), as an alternative scaffold to Matrigel^®^ for culturing 3D salivary gland cells. A protocol for an optimized EWA was established by comparing cell viability using 1%, 2%, and 3% alginate solution. The normal salivary simian virus 40-immortalized acinar cell (NS-SV-AC) and the submandibular gland-human-1 (SMG-hu-1) cell lines were also used to compare the spheroid formation and cell viability properties of both scaffold biomaterials; cell viability was observed over 10 days using a Live–Dead Cell Assay. Cell viability and spheroid size in 2% EWA was significantly greater than 1% and 3%. It is evident that EWA can support salivary cell survivability as well as form larger spheroids when compared to cells grown in Matrigel^®^. However, further investigations are necessary as it is unclear if cultured cells were proliferating or aggregating.

## 1. Introduction

Salivary glands are characterized as exocrine saliva-secreting tissues that reside throughout the oral cavity. Human salivary glands consist of three major pairs: the submandibular glands, sublingual glands, and parotid glands [1,2]. Additionally, 600–1000 minor salivary glands can be found throughout the oral cavity, namely, the buccal, labial, distal palatal, and lingual mucosal regions and pharynx [2]. Together, these salivary glands function to secrete saliva which in turn aids in lubricating the oral cavity, digesting food, maintaining homeostasis of oral cavity, providing microbial protection, and remineralizing teeth [3,4]. Because saliva plays such a crucial role in the oral cavity, complete or significant reduction in saliva production can lead to a condition known as xerostomia, also known as dry mouth, which will have devastating effects in the mouth and one’s quality of life. The main etiology of xerostomia are adverse effects from medication for other diseases and conditions, radiotherapy for head and neck cancer (HNC) patients, and those with Sjörgren’s Syndrome. HNC patients and individuals with Sjörgren’s syndrome have their salivary glands destroyed as a result of cytotoxic doses of radiation and autoimmune attacks on the glands, respectively [5,6]. Annually, there are approximately 500,000 new cases of HNC and 75,000 new cases of primary Sjörgren’s Syndrome worldwide [4,7]. Those that suffer with xerostomia typically also experience difficulty in speech, mastication and swallowing, taste loss, and have increased susceptibility to dental caries and oral infections and diseases [4]. Furthermore, these patients only have palliative treatments, e.g., frequent water sipping, using gel or spray saliva substitute, and/or taking saliva-stimulating drugs such as pilocarpine hydrochloride or cevimeline hydrochloride. [8,9]. The high prevalence of xerostomia in addition to the lack of treatment options highlight the urgent need for research and development towards a curative treatment.

One branch of research that contributes toward the development of a curative treatment is biomaterial development, which include the scaffolds being used to culture cells in 3D. Optimizing these scaffolds can result in cells, spheroids, or organoids grown with phenotypes and functions that more closely resemble their normal biological counterparts. This would ultimately facilitate more reliable and reproducible results when using these cells in other studies such as drug therapy testing, disease modeling and analysis, and genetic screening and editing. There are many factors to be considered when developing an ideal biomaterial scaffold, which varies between cell types as many factors including essential nutrients, physical properties, anchorage points, growth factors, hormones, and vitamins, all contribute to specific developmental outcomes [10,11,12]. However, three fundamental components of organogenesis which require higher attention, are the type of cells in the system, the growth factors that guide and nurture the growth of the cells, and the scaffold hosting the cells. Together these components are known as the triad of tissue engineering; optimizing each fundamental component improves the outcome for successful organogenesis [13]. The cells in proximity to each other can have influence on cell morphogenesis, development, and differentiation through cell–cell interaction. In a study by Nogawa and Mizuno (1981), they showed that the presence of mesenchymal cells can influence quail salivary epithelial cells to elongate and branch [14]. In a study by Patel et al. (2006), it was suggested that even neuronal cells can impact salivary gland cell branching and development [15]. Cellular activities can also be influenced by growth factors. The presence and binding of growth factors to cell surface receptors can induce changes in cellular metabolic activity, migration, differentiation, proliferation, morphogenesis, and survival [16]. It is known that specific cells require specific growth factors to grow and differentiate, e.g., neurocytes require the addition of nerve growth factors (NGF), keratinocytes require epidermal growth factors (EGF), and endothelial cells and fibroblasts require basic fibroblast growth factors (bFGF) [17]. Cell attachment is a major function of the final fundamental component to prioritize in organogenesis—the scaffold. A scaffold used for tissue engineering is typically composed of some biocompatible material and serves to protect the cells, act as a template for tissue formation, and form a specific microenvironment that is niche to the tissue of interest which may include specific temperatures, oxygen and carbon dioxide saturation level, pH level, stiffness/compliance of surrounding tissue, osmolality, attachment substrates, and availability of nutrients and growth factors [13,18,19]. An ideal candidate scaffold should also be: (1) degradable, ideally at the rate of tissue regeneration; (2) biocompatible, which means the biomaterial or its degradation product does not illicit an immune reaction and allows normal cell migration and integration; (3) mechanically tunable, for example, in shape, stiffness, porosity, etc.; and (4) cost-effective [13,20].

Matrigel^®^ is a hydrogel composed of extracellular proteins including collagen IV, laminin, fibronectin, entactin, and perlecan, and growth factors which are derived from Engelbreth-Holm-Swarm mouse sarcoma cells. It is often considered as the gold standard in 3D cell culture research due to its ability to accommodate cell attachment, morphogenesis, proliferation, and organization [21,22]. While many studies have successfully grown and studied 3D salivary cells using Matrigel^®^, its disadvantages are that it is expensive to use, has a quick degradation rate, and is potentially tumorigenic and/or immunogenic [22]. As a result, there is a need for a more biocompatible and accessible alternative for 3D cell culture scaffolds.

This paper explores the use of a novel hybrid hydrogel, Egg White-Alginate (EWA). In recent years, egg white (EW), a protein-dense substance, demonstrated excellent capability in supporting cellular attachment, differentiation, and survivability when being used as a scaffold. The egg white component is mainly composed of ovalbumin (>50%); however, it also contains other ECM-resembling proteins that can act as a substrate for cellular attachment [23,24]. In a study by Kaipparettu et al. (2008), the use of egg whites as a scaffold to culture epithelial breast tumor cells, demonstrated it has similar cell growth and differentiation capabilities as those grown on Matrigel^®^ [23]. Similar observations have also been noted in another study comparing egg white and Matrigel^®^ in culturing human umbilical vein endothelial cells [24]. Alginate is also a very commonly used biomaterial due to its versatile and tunable properties. The stiffness, elasticity, compressibility, viscoelasticity, degradation rate, and shape, among other physical properties can easily be manipulated [25]. By combining these two biomaterials, it provides researchers with an extremely affordable and accessible alternative to culture cells in 3D. This pilot study aimed to establish a protocol for creating Egg White-Alginate and to explore the feasibility of using Egg White-Alginate as a biomaterial scaffold to culture salivary gland cells in 3D.

## 2. Materials and Methods

### 2.1. Scaffold Development

#### 2.1.1. Egg White Isolation and Heat Treatment

Fresh eggs (large white eggs (omega-3)) were purchased from a local grocery store in Montreal (QC, Canada). The external surfaces of the eggs were disinfected with 70% ethanol. The egg is gently cracked and a perforation with approximately 1 cm diameter is created. The egg white component was poured into a 50 mL conical centrifuge tube using forceps; all other contents (egg shell, chalaza, yolk, and watery content) were discarded. The tubes were then placed in an incubator at 58 °C for 1 h to sterilize (pasteurize).

#### 2.1.2. Alginate Preparation

Sodium alginate, low molecular weight solutions (398.31 g/mol) (Protanal LF 5/60, FMC BioPolymer, Philadelphia, PA, USA) were prepared by slowly dissolving a small amount of sodium alginate powder into a 50 mL conical tube containing a solution of 1:3 Hank’s Balanced Salt Solution (Gibco 14025076 ON, Canada)/Epi Max (Wisent Bio Products, 002-010-CL, QC, Canada). Between each addition of powder, the solution was vigorously mixed by manually shaking for a 5 s and pulse-vortexing 5–10 times. Once the desired alginate solution concentration was achieved (1%, 2%, and 3% by weight), the tubes were then placed on a Speci-Mix Aliquot Mixer (Thermolyne, M71015) in a 37 °C incubator for 30 min to further mix and dissolve the alginate and to eliminate any clumps that were formed.

#### 2.1.3. Egg White-Alginate Hydrogel Preparation

EWA hydrogels were created by combining 2 parts egg white and 1 part sodium alginate into a 50 mL conical tube. The mixture was pipetted vigorously to mechanically break the egg white until the mixture was homogenous. The homogenized mixture was then centrifuged at 300 G for 90 s at 4 °C to separate bubbles from the mixture; the foam layer was isolated and discarded. A trimmed micropipette tip was then used to plate approximately 1 mL, 300 μL, and 100 μL of EWA into each well of a 6-well plate, 24-well plate, and 96-well plate, respectively. Wells containing EWA were crosslinked with a 1% calcium chloride (CaCl_2_) in double distilled water crosslinking solution by slowly suspending 3 parts crosslinking solution for each part EWA. The plates are then placed into a 37 °C and 5% CO_2_ incubator overnight; the CaCl_2_ solution was aspirated and discarded the following day. The EWA hydrogel scaffold was covered with culture medium and stored in the incubator when not in use.

#### 2.1.4. Matrigel^®^ Preparation

Matrigel^®^ (Corning, C356234) was thawed overnight in an ice bath within a 4 °C refrigerator. Frozen pipette tips were placed in a −20 °C refrigerator overnight as well. Matrigel^®^ was transferred into a 15 mL conical centrifuge in an ice bath using cold tips and then diluted with 5 parts ice-cold cell culture media. The solution was homogenized by repeated pipetting and pulse-vortexing. Then, 1 mL, 300 μL, and 100 μL Matrigel^®^ solution were distributed into wells of a 6-well plate, 24-well plate, and 96-well plate respectively. The plates containing Matrigel^®^ were placed into a 37 °C and 5% CO_2_ incubator to stiffen for 1 h. Throughout the manipulation, all plates were rested on ice packs, cold tips were used, and Matrigel^®^ and conical tubes rested in an ice bath.

### 2.2. Physical Property Tests

#### 2.2.1. Determining Optimal Alginate Percentage

Either 1%, 2%, or 3% EWA scaffolds were created and individually crosslinked in separate wells of a 6-well plate. Each well was then seeded with 50,000 NS-SV-AC cells on top of the scaffold and was then submerged in Epi Max growth media and stored in an incubator at 37 °C and 5% CO_2_ for 5 days. Random bright-field images were taken via light microscopy (Leica, DM IL) on day 5 at 50× and 200× magnifications, in triplicates. The diameter of the 5 largest spheroids in each image were recorded and averaged to obtain the average spheroid diameter size from each treatment.

#### 2.2.2. Determining Degradation Rate

Two percent EWA scaffolds were created and individually crosslinked in each well of a 24-well plate. Each well was then seeded with 70,000 NS-SV-AC cells on top of the scaffold and was then submerged in Epi Max growth media which was refreshed every 3 days. The degradation rate of the scaffold was determined by weighing the solid mass of the scaffold over 30 days.

### 2.3. Biological Tests

#### 2.3.1. Cell Viability

Sixty-two percent EWA scaffolds were created and individually crosslinked in each well of a 6-well plate. Either 150,000 NS-SV-AC or SMG-hu-1 cells were seeded on top of the hydrogel for each well and then submerged in Epi Max growth media which was refreshed every 3 days; cells were grown for 15 days in an incubator at 37°C and 5% CO_2_. Live–Dead staining was performed on day 5 and 10 using a Live and Dead Cell Assay kit (Abcam, ab115347). The 4 mM calcein acetoxymethyl (CalAM) and 2mM ethidium homodimer III (EthD-III) stock solutions were diluted with 1X PBS to create a working solution of 2 μM for each stain. Cells were stained with both CalAM and EthD-III simultaneously, covered with tin foil, and incubated at room temperature for 1 h. Bright-light and fluorescent images were taken using the Leica DM IL microscope in the dark at 50x magnification.

#### 2.3.2. Spheroid Formation

Two percent EWA and Matrigel^®^ were plated in each well on separate 6-well plates. Either 150,000 NS-SV-AC or SMG-hu-1 cells were seeded on top of the hydrogel for each well and then submerged in Epi Max growth media which was refreshed every 3 days; cells were grown for 20 days in an incubator at 37 °C and 5% CO_2_. Bright-field images were taken at random using the Leica DM IL microscope on day 1, 2, 5, 10, 15, and 20 at 50×, 100×, and 200× magnifications. Once taken, the diameter of the 5 largest spheroids in each image were recorded and averaged to obtain the average spheroid diameter size from each treatment.

### 2.4. Statistical Analysis

Statistical analysis was conducted using the GraphPad Prism 6 package (GraphPad Software Inc., CA, USA). All test samples were performed in triplicate. A repeated measure (RM) one-way ANOVA, multiple comparison test with the Greenhouse–Geisser correction was used to analyze values between groups; a RM two-way ANOVA with Sidak’s multiple comparisons test was used to compare values between groups over time. A *p*-value < 0.05 was considered statistical significance.

## 3. Results

### 3.1. Scaffold Development

**Figure 1:**
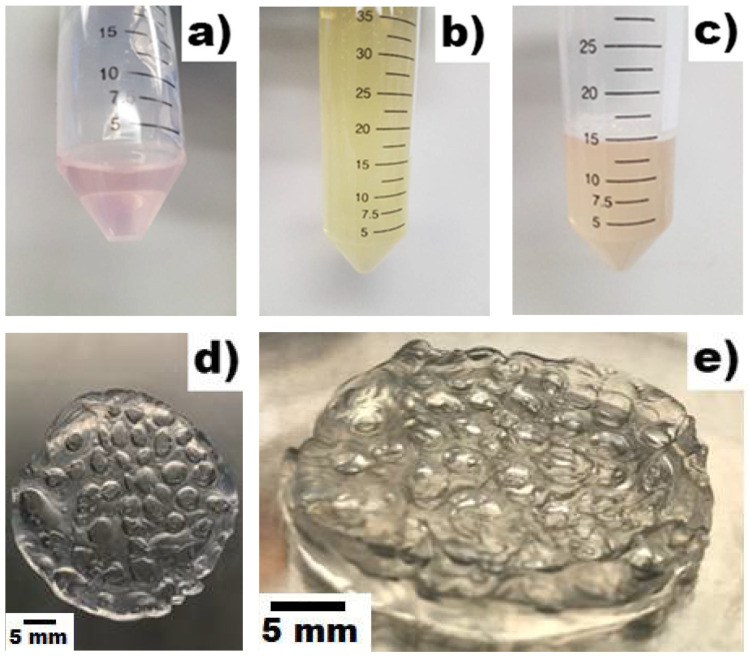
Reference images for EWA protocol: (**a**) Image of alginate (2%) dissolved in Epi Max medium. (**b**) Image of extracted EW. (**c**) Image of egg white and alginate mixed together post-centrifugation forming froth layer on top. (**d**) Image of EWA from top view, illustrating the air bubbles and craters created post-crosslinkage. (**e**) Close image of EWA to illustrate the topography, texture, and thickness of the scaffold post-crosslinkage.

Following the EWA scaffold development protocol, the EWA hydrogel had a flexible aloe-like texture and appearance. The scaffolds also had craters where the crosslinking solution was suspended, and small air bubbles dispersed throughout the hydrogel (Figure 1). It is important to note that during the mixing phase, air bubbles are inevitably incorporated into the hydrogel due to vigorous shaking and mixing. However, ensuring adequate homogenization between the egg white and alginate components took precedence over the formation of air bubbles. While these bubbles can interfere with microscopic photos if left not removed prior to cross-linking, light centrifugation (300G × 90 s) can minimize interference by separating the air bubbles from the solution (Figure 1c).

### 3.2. Physical Tests

#### 3.2.1. Optimal Alginate Percentage

**Figure 2:**
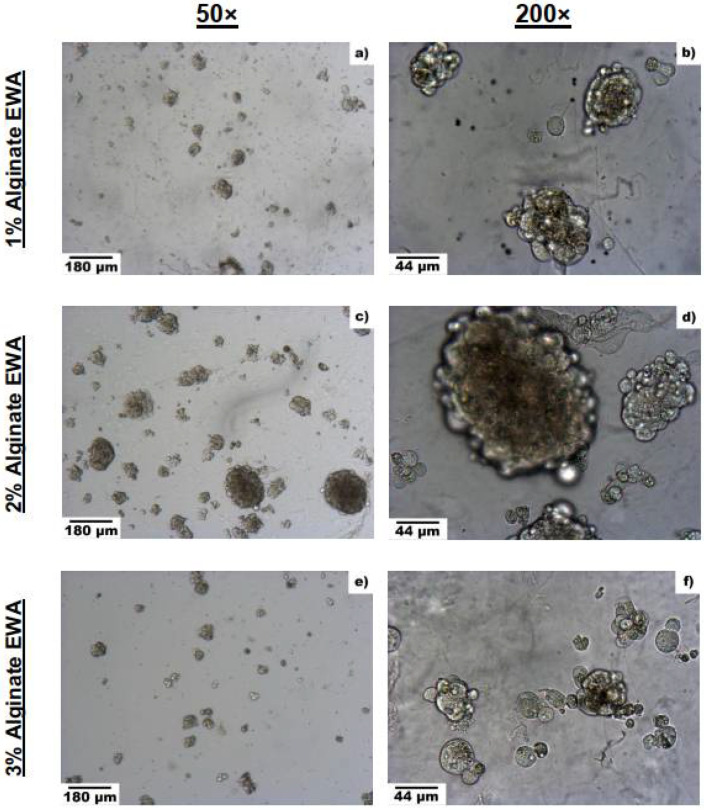
Images of NS-SV-AC grown on EWA at 1%, 2%, and 3% alginate on day 5: Images (**a**,**b**) represent NS-SV-AC grown on 1% alginate EWA, taken at 50× and 200× respectively. Likewise, images (**c**,**d**) represent NS-SV-AC grown on 2% alginate EWA at 50× and 200×, and images (**e**,**f**) represent NS-SV-AC grown on 3% alginate EWA at 50× and 200×.

**Figure 3:**
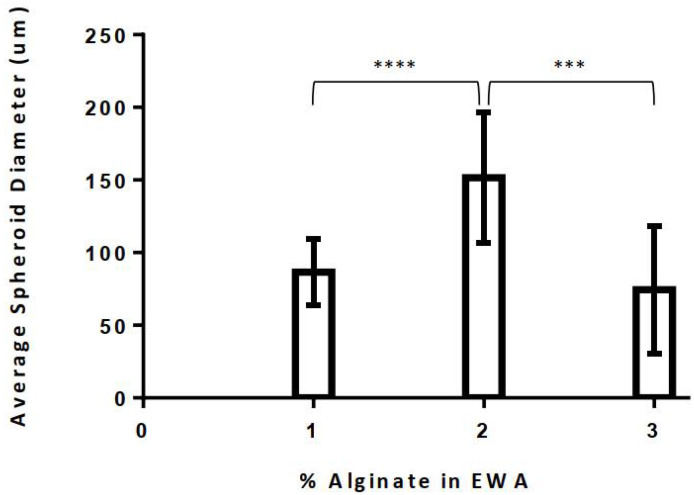
Comparison of spheroid sizes on different alginate concentrations for EWA hydrogel. The value of each bar shown was reported as a mean where *n* = 15 for each group. Sample images were taken at random, where the five largest spheroids within each image were identified and had its diameter measured. The average spheroid diameter size from each treatment was then reported. Statistical significance was analyzed using a RM one-way ANOVA, multiple comparison test with the Greenhouse–Geisser correction, where *** represents *p* < 0.001%, and **** represents *p* < 0.0001%. The error bars shown represent the SE of each group.

After establishing the protocol for creating EWA, cells were then seeded on top of the EWA scaffold and submerged with cell media. Various tests were then conducted to optimize and characterize the novel biomaterial. First, the cell culturing capability of the hydrogel was examined based on the alginate composition percentage. EWA was produced using a 1%, 2%, or 3% alginate solution to observe changes in cellular behavior; on day 5, images of NS-SV-AC cells were taken at 50× and 200× magnification (Figure 2). Regardless of alginate percentage, it is evident that EWA can support NS-SV-AC cells after 5 days. However, a visual comparison in spheroid formation across the varying alginate percentages suggested that more cells were present and formed larger spheroids in the 2% alginate EWA group compared to the 1% and 3% groups. When comparing spheroid sizes across the groups, cells plated on the 2% alginate EWA scaffold formed significantly larger spheroids compared to those cultured on the 1% (*p* < 0.0001) or 3% (*p* < 0.001) alginate EWA group (Figure 3). It was also found that the absolute largest spheroid sizes (in diameter) were present in the 2% group compared to the 1% and 3% alginate group. However, it is unclear if the cell density and spheroid formation size was a result of cell migration and aggregation or was due to cellular proliferation. This test provided evidence that a 2% alginate EWA hydrogel would be best for culturing NS-SV-AC in 3D.

#### 3.2.2. Degradation Rate

**Figure 4:**
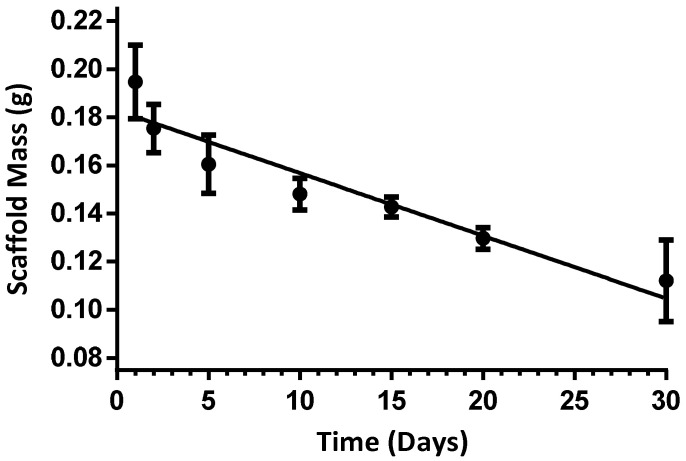
Change in mass of 2% alginate EWA plated with NS-SV-AC over time. Table 0. x + 0.1828. The data is presented as a mean, where the error bars shown represent the SE. *n* = 3 at each time point.

The degradation rate of the 2% alginate EWA scaffold was examined over 30 days. The degradation rate was determined by examining the change in scaffold mass over time. It was calculated to be approximately −0.002603 g/day (Figure 4).

### 3.3. Biological Tests

#### 3.3.1. Cell Viability

**Figure 5:**
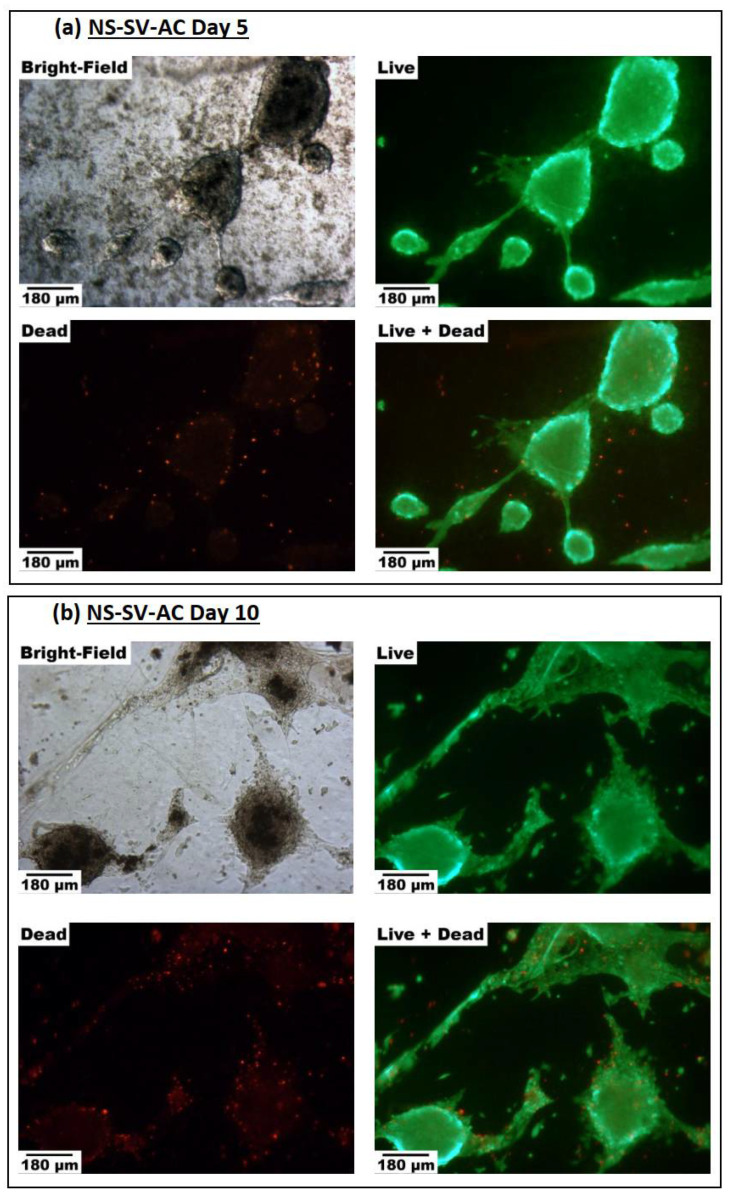

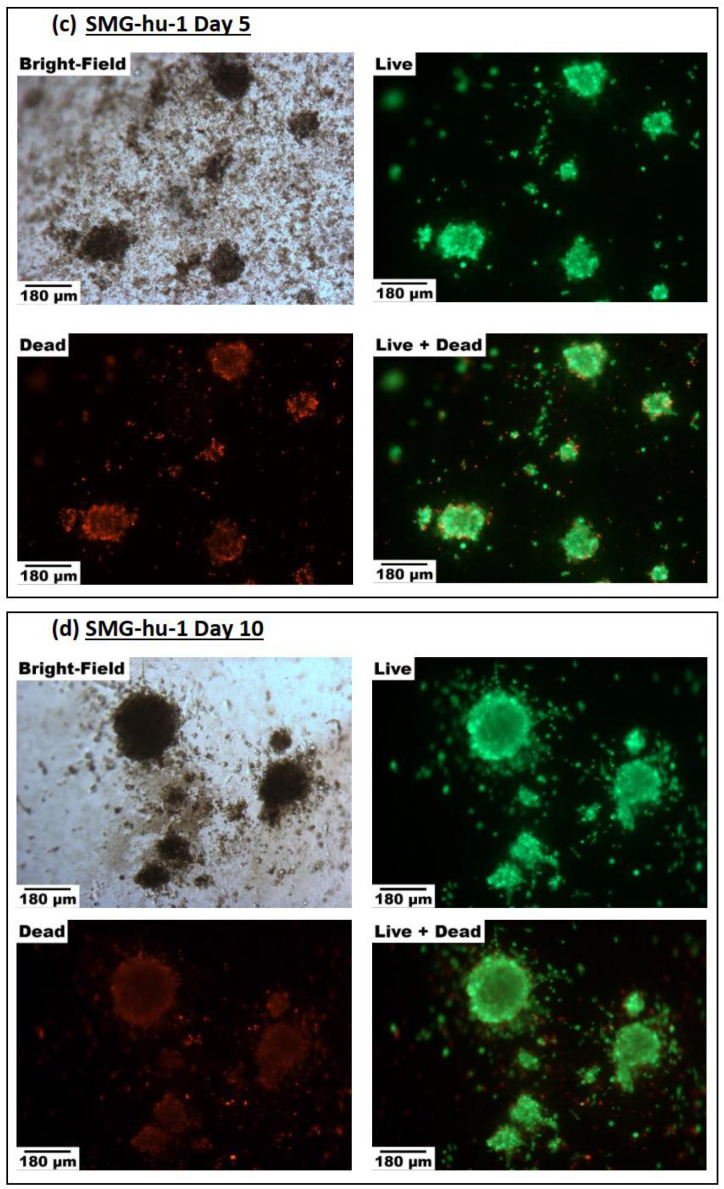
(**a**–**d**). Bright-light and Fluorescent Live–Dead images of salivary gland cells grown on EWA. Each figure (**a**–**d**) contains as set of images capturing either NS-SV-AC cells or SMG-hu-1 cells on day 5 or day 10, under bright-field light, fluorescent light with CalAM Live stain, fluorescent light with EthD Dead stain, and a combined superimposed image of the Live–Dead stained cells. (**a**) displays NS-SV-AC cells on day 5. (**b**) displays NS-SV-AC cells on day 10. (**c**) displays SMG-hu-1 cells on day 5. (**d**) displays SMG-hu-1 cells on day 5. Images were taken at 50× magnification; the scalebar on the bottom left corner represents 180 μm.

NS-SV-AC and SMG-hu-1 cells were stained with CalAM (live) and EthD-III (dead), and then observed under fluorescent light to determine the feasibility of the EWA as a scaffold for culturing salivary gland cells over 10 days. The persistent fluorescent green stain evident across day 5 to day 10 in both salivary cell lines suggests that at minimum, cells are capable of surviving on the EWA scaffold. With regards to the fluorescent red-stained cells, which represent dead cells, there are visually fewer dead cells than there are live green cells. Additionally, there is a visually comparable number of florescent dead cells between day 5 and day 10 in both salivary cell line. It is also evident that cells are forming 3D spheroids over time; however, it is unclear whether these spheroids are being formed through cell proliferation, cell aggregation, or a combination of both. Together, these images suggest that EWA can serve as a feasible scaffold in supporting salivary cell life for at least 10 days. It is also noteworthy that NS-SV-AC cells tend to form these linear structures that branch out from the spheroids as evident in Figure 5a,b. However, these branching structures are absent in the SMG-hu-1 cell line (Figure 5c,d).

#### 3.3.2. Spheroid Formation

**Figure 6:**
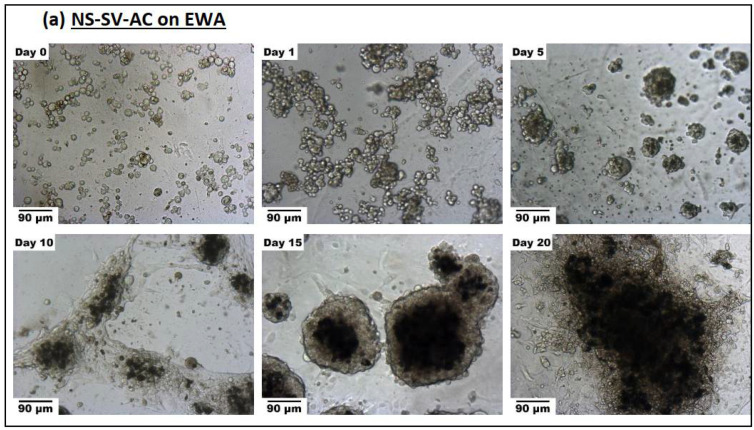

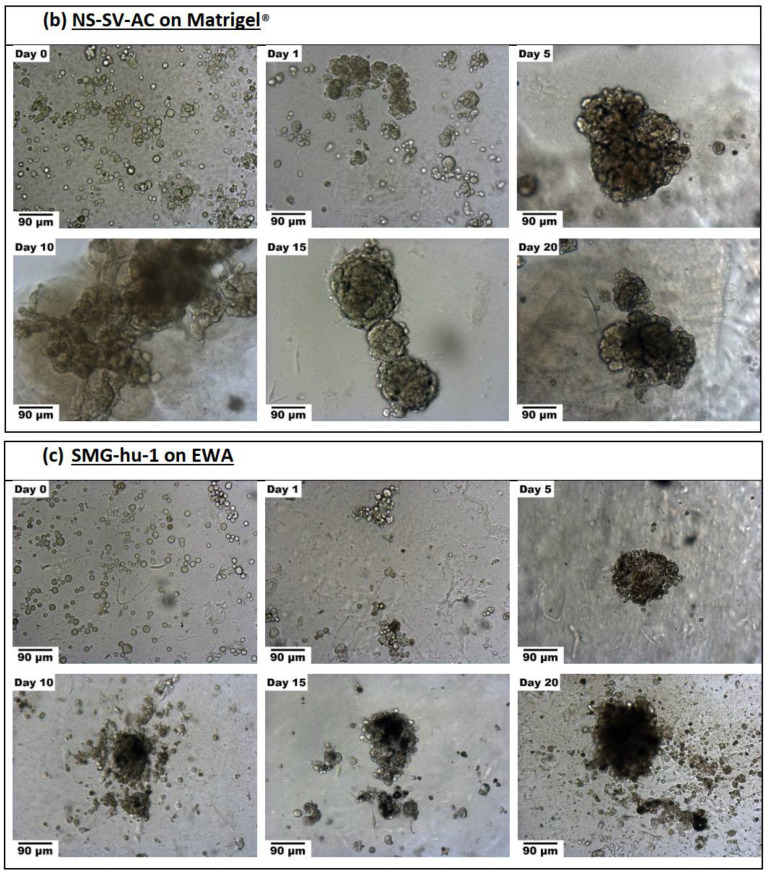

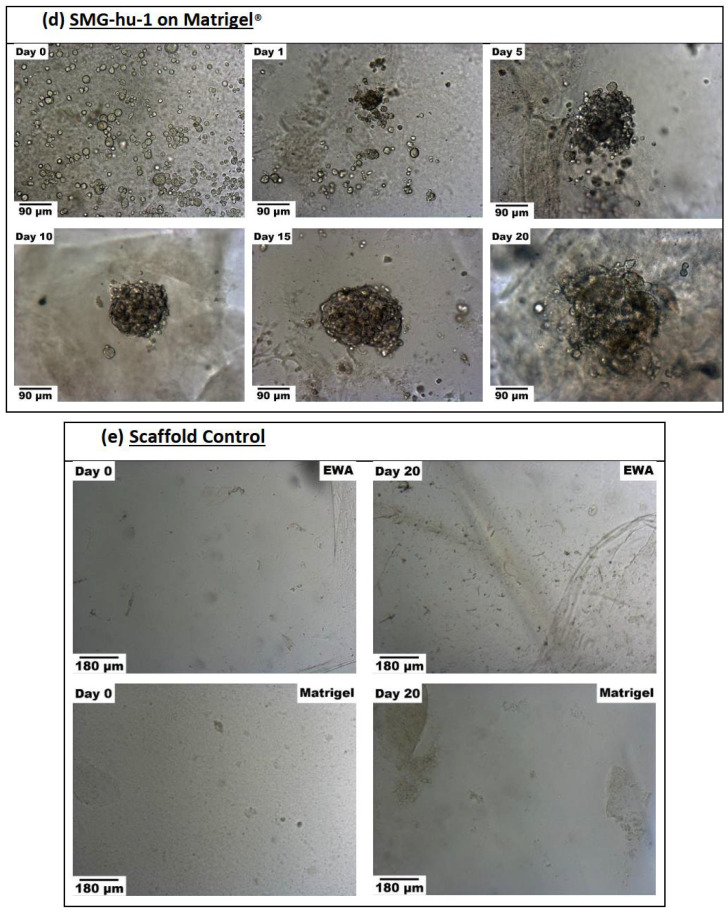
(**a**–**e**). Comparison of progression in cell growth and spheroid formation of cells grown on EWA and Matrigel^®^: (**a**) NS-SV-AC cells grown on EWA. (**b**) NS-SV-AC grown on Matrigel^®^; (**c**) SMG-hu-1 cells grown on EWA; (**d**) SMG-hu-1 cells grown on Matrigel^®^; (**e**) EWA and Matrigel^®^ grown without any cells. Each group (**a**–**d**) contains a chronological series of photos taken on day 0, 1, 5, 10, 15, and 20. All images were taken with a bright-light microscope at 100× magnification and at random. The black scalebar on the bottom left corner of every image in Figure 6a–d represents 90 μm; the scale bar on the bottom left corner of images in Figure 6e represents 180 μm.

**Figure 7:**
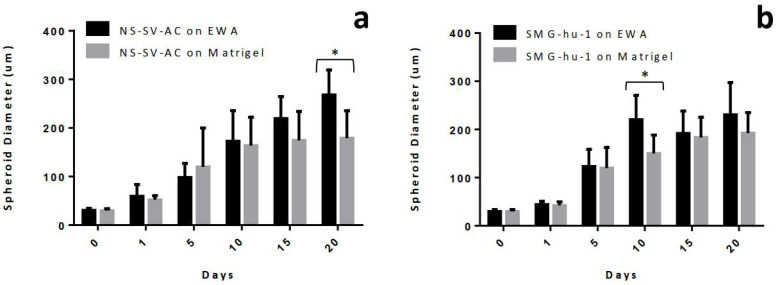
Comparison of average spheroid sizes (in diameter) of cells grown on EWA or Matrigel^®^: (**a**) compares NS-SV-AC across EWA and Matrigel^®^ scaffolds. (**b**) compares SMG-hu-1 across EWA and Matrigel^®^ scaffolds. The value of each bar shown was reported as a mean where *n* = 15 for each group. Statistical analyses were performed using a RM two-way ANOVA with Sidak’s multiple comparisons test; * *p* < 0.05. Data are presented as mean ± standard error (SE). The error bars represent the SE of each measurement.

Both NS-SV-AC and SMG-hu-1 cell lines were grown on EWA and Matrigel^®^ scaffolds to analyze growth and phenotypic differences in these salivary cells across the two scaffold types. Images shown in Figure 6a–d present cells grown on top of either EWA or Matrigel^®^; though over time, these cells can be found on top of the gel and as well as suspended throughout the thickness of both hydrogels. When comparing NS-SV-AC grown on EWA and Matrigel^®^, it is evident that both scaffolds led to spheroid formation over 20 days (Figure 6), though it is uncertain as to whether the formation of spheroids are due to cellular proliferation or due to cell migration and aggregation. However, it is more likely that the formation of spheroids resulted from the migration and aggregation of the single cells as the number of single cells decreased while the average spheroid size (in diameter) increased throughout the duration of the experiment. A statistical analysis reveals significant evidence (*p* < 0.0001) for spheroid formation over 20 days in all groups. When comparing cells grown on EWA (Figure 6a,c) versus cells grown on Matrigel^®^ (Figure 6b,d), the average spheroid sizes were found to be larger in the EWA group on day 20 for NS-SV-AC (Figure 7a) and day 10 for SMG-hu-1 (Figure 7b). Additionally, spheroids in the EWA group exhibited dark cores on day 10, 15, and 20, which was not as prominent in the Matrigel^®^ group. It could be possible that these dark central features are local high-density clusters of cells. It is unclear as to why these features are present in the EWA group but not the Matrigel^®^ group. Another notable difference between the NS-SV-AC grown on EWA and Matrigel^®^ is that those grown on the former had visually distinct features throughout the scaffold. For example, the image captured on day 5 and 20 shows the NS-SV-AC growing out laterally rather than clustering together to form spheroids as seen on day 5 and 15. While not shown, these different features were not time dependent as both features can be found across all time points. It was also noted that the SMG-hu-1 cell line has lower potential to grow in 3D than the NS-SV-AC cell line. While plated in equal density across all four groups, it was evident that the total number of spheroids and single cells remaining in the culture were lower in the SMG-hu-1 groups compared to the NS-SV-AC group, regardless of the scaffold. Additionally, there was a visual difference between the average spheroid sizes of SMG-hu-1 and NS-SV-AC with the former being smaller. Images of scaffolds without cells were taken to visualize physical changes in the scaffold, and its possible influences, interference, and distortion on cell imaging (Figure 6e).

Overall, this experiment provided a visual comparison between cells grown on EWA and Matrigel^®^. This study showed evidence for maintaining cell growth and spheroid formation. Additionally, this study confirmed that there are morphological differences in cells grown on different scaffolds, and even within scaffolds, depending on its physical properties. While this experiment showed evidence of spheroid formation of salivary cells over 20 days—highlighting the potential use of EWA as an alternative scaffold for salivary cell culture—further biological studies are needed to provide further information and evidence for the use of EWA as scaffold, particularly regarding the impact of certain EWA scaffold characteristics on cell morphology, differentiation, function, migration, survivability, and growth.

## 4. Discussion

Throughout this study, we were able to display various advantages and traits of using egg white alginate as an alternative to Matrigel. The slower degradation rate of EWA provides researchers with the advantage of performing longer-term studies. Additionally, its more robust characteristics enable researchers to follow specific population of cells on the scaffold throughout the duration of the experiment, with minimized risk of losing track of specific cell groups. The results in our study also suggest that EWA is a feasible scaffold for salivary cell culture. Relative to Matrigel, our study showed that EWA is both capable of culturing larger spheroids and for longer due to a slower degradation rate. However, because this is a pilot study exploring novelties of EWA as a scaffold, there remain unknowns that require future studies to confirm and reinforce our findings. For example, while our results show that EWA is capable of culturing larger spheroids, the effects of EWA on cellular gene and protein expression and regulation, cell interaction and organization, and morphological and functional changes are not known. Future studies could aim to monitor specific key protein expression such as AQP-5 throughout the duration of the study to determine changes in function.

In the spheroid formation experiment, morphological differences in NS-SV-AC are observed throughout the duration. These differences in NS-SV-AC behavior could be attributed to inconsistencies in scaffold roughness and stiffness. The technique used to create the hydrogel involves manual pipetting and pulse-vortexing egg white with an alginate solution, which may not adequately homogenize the EWA hydrogel. Perhaps a more homogenous blend between the egg white and alginate component could lead to more consistent cell aggregation behavior throughout the entire scaffold and across samples. In a study by Zhang et al., they found that cells grown in 0.8% alginate scaffolds tend to have a 3D interconnected cellular network similar to that exhibited in Figure 6a Day 10, while cells grown in 1.8% and 2.3% alginate scaffolds exhibited spheroid-like structures similar to that exhibited in Figure 6a Day 5 and 15 [26]. Another factor that could be affecting the varied cell morphology is whether cells are on top of the hydrogel or suspended within the hydrogel. Due to the difference in stiffness between egg white and alginate, cells suspended in areas that are potentially more abundant in alginate may result in cells sinking further into the hydrogel, despite being initially plated on top of the hydrogel.

The main limitation to our study and use of EWA as a scaffold is that further research needs to be conducted to confirm biological mechanisms such as the interactions between egg white proteins and salivary cell receptors. As previously mentioned, it is also imperative for future studies to determine changes in protein expression, morphology, cell interaction, and function. Lastly, future studies should also aim to further characterize the scaffold, e.g., elastic modulus and surface roughness, which would ultimately allow us to understand the nature of the gel and its impact on cellular activity. Further understanding of these various aspects will enable further optimization of the novel hydrogel. However, because EWA is a novel hydrogel, there is a lack of pre-established protocols for isolating cells to perform certain studies and tests such as immunofluorescent staining, DNA isolation, and measuring elastic modulus. Thus, the first hurdle to overcome is to establish effective protocols for manipulating the scaffold in the presence and absence of cells.

While this study reports the first development, use, and experimental results of EWA, our team has previously published a follow-up study by Zhang et al. (2020) addressing an optimized protocol for producing a smoother EWA using frozen CaCl_2_ disks to crosslink the EWA hydrogel [27]. It is important to address the discrepancy in our results between the two studies; while this study reports that 2% alginate EWA had significantly larger average spheroid sizes (Figure 3) when compared to 1% and 3% alginate EWA, our 2020 study reported having the largest spheroid sizes in the 3% alginate EWA group. This discrepancy could be contributed to difference in EWA creation protocol. In this study, EWA was crosslinked using drops of liquid CaCl_2_ which caused large crater-like features on contact (Figure 1e). This was a limitation of our initial protocol as the crater features could not be controlled and standardized across sample sizes and studies. These craters could result in an uneven distribution of cells across the scaffold due to gravity and random chance. In our 2020 study, by using frozen CaCl_2_ disks, we were able to minimize the impact of the CaCl_2_ solution droplets, forming a smoother and more uniform surface; thus, cells could be plated and distributed on the scaffold more uniformly [27]. Consequently, the results from that study likely reflect a more accurate representation of the impact of various alginate concentrations in EWA on cell viability and spheroid formation. However, more consistently with this study’s data, our 2020 study determined that 3% alginate EWA had lower cell proliferation and viability relative to other samples, with 1.5% alginate EWA having the highest viability. While we did not test 1.5% alginate EWA in this study, our study does suggest that an alginate concentration of 2% visually revealed higher cell viability (Figure 2). This poses a challenge as there seems to be a delicate balance between scaffold stiffness, cell viability, and spheroid formation. Future studies should aim to closely monitor scaffold physical properties and its biological effect on salivary cell activity.

## 5. Conclusions

In this study, we established an initial protocol for developing and plating EWA, which was optimized at 2% alginate concentration. At this concentration, there was a significant difference in cell growth and survival, relative to cells grown on EWA composed of 1% or 3% alginate concentrations. We demonstrated that EWA is capable of maintaining two different salivary cell lines for 20 days. We also demonstrated that salivary cells grown on EWA led to spheroid formation, similar to that of Matrigel. The future direction of our study is to establish effective protocols for manipulating the scaffold and isolating cells from the scaffold, further characterize the physical and biological properties of the hydrogel, closely examine cellular changes in cells grown on the hydrogel, and attempt to culture human primary salivary cells on EWA (rather than immortalized cell lines). These are the next goals and steps to further proving the use of EWA as an alternative to Matrigel for the use of salivary cell culturing. These salivary cells should ideally resemble primary human salivary cells, thus bringing EWA one step closer to being a feasible and biologically relevant scaffold with clinical applications such as in drug-screening and disease modeling.

## Data Availability

Data is contained within the article.
